# The Puzzle of Hereditary Spastic Paraplegia: From Epidemiology to Treatment

**DOI:** 10.3390/ijms23147665

**Published:** 2022-07-11

**Authors:** Arun Meyyazhagan, Haripriya Kuchi Bhotla, Manikantan Pappuswamy, Antonio Orlacchio

**Affiliations:** 1 Dipartimento di Medicina e Chirurgia, Università di Perugia, 06132 Perugia, Italy; arun47biotech@gmail.com; 2 Department of Life Sciences, CHRIST (Deemed to be University), Bengaluru 560029, India; hpriya9121@gmail.com (H.K.B.); manikantan.p@christuniversity.in (M.P.); 3 Laboratorio di Neurogenetica, Centro Europeo di Ricerca sul Cervello (CERC), Istituto di Ricovero e Cura a Carattere Scientifico (IRCCS) Fondazione Santa Lucia, 00179 Rome, Italy

**Keywords:** hereditary spastic paraplegia, genes, pathways, drugs, therapies, clinical manifestations

## Abstract

Inherited neurodegenerative pathology characterized by lower muscle tone and increasing spasticity in the lower limbs is termed hereditary spastic paraplegia (HSP). HSP is associated with changes in about 80 genes and their products involved in various biochemical pathways, such as lipid droplet formation, endoplasmic reticulum shaping, axon transport, endosome trafficking, and mitochondrial function. With the inheritance patterns of autosomal dominant, autosomal recessive, X-linked recessive, and mitochondrial inheritance, HSP is prevalent around the globe at a rate of 1–5 cases in every 100,000 individuals. Recent technology and medical interventions somewhat aid in recognizing and managing the malaise. However, HSP still lacks an appropriate and adequate therapeutic approach. Current therapies are based on the clinical manifestations observed in the patients, for example, smoothing the relaxant spastic muscle and physiotherapies. The limited clinical trial studies contribute to the absence of specific pharmaceuticals for HSPs. Our current work briefly explains the causative genes, epidemiology, underlying mechanism, and the management approach undertaken to date. We have also mentioned the latest approved drugs to summarise the available knowledge on therapeutic strategies for HSP.

## 1. Introduction

Hereditary spastic paraplegia (HSP) is an amalgamation of inherited single-gene neuronal degenerative and developmental diseases with significant clinical manifestations, such as spasticity and fragility, predominately in the lower extremities [1]. Other pathological manifestations include degenerating descending fibers in the corticospinal and posterior columnar regions in either autosomal dominant, autosomal recessive, mitochondrial, or X-linked inheritance patterns [2]. HSP pathologies are designated as spastic paraplegia genes (SPG); to date, 80 SPGs have been identified with a rare prevalence of 1–5 in 100,000 individuals varying around the globe. According to a meta-analysis, the global prevalence of SPG is 1.8/100,000, with SPG3A, SPG4, and SPG11 subtypes predominating in registered cases and being infrequently diagnosed in consanguineous settings [3,4]. A meta-analytic study comprising 13,570 individuals showed the frequently recurring genetic alteration in *SPAST*, *REEP1*, *ATLL1*, SPG11, SPG15, SPG7, SPG35, SPG5, and SPG54. *SPAST* is observed in 25% of the autosomal dominant cases, followed by *REEP1* and *ATL1*. SPG11 was observed in highly recessive patterns with varying prevalence rates [5].

Clinically, HSP is distinguished as pure and complex based on the presence of any add-on neurological manifestations other than paraparesis, as described by Harding [6]. HSP progression is slower due to the neural dysfunction in the corticospinal region. HSP includes complications such as losing vibration senses (abnormal sensory pathway) and loss of bladder control [1]. However, complex HSP is accompanied by neurological and non-neurological manifestations, such as ataxia, epilepsy, neuropathy, optic atrophy, dysarthria, and skeletal deformities [1,7]. HSP lacks a straightforward genotypic-phenotypic association due to the sudden onset of manifestations irrespective of age. The symptoms can be triggered as early as infancy or late adulthood with various clinical presentations at different progressive and disability rates within the same families with similar mutations [8].

Advancing technologies such as next-generation sequencing have paved the way for creating genetic panels for the most rare diseases, including HSP. However, due to the lack of suspected cases, the molecular diagnosis is limited [9]. Though advancing genetic testing has increased the identification of patients in the past few years, managing and determining is still challenging. The current era focuses on providing the benefits of using gene editing methodology as personalized drugs in patients with specific genotypes. This is not possible with HSP as the drug should target 80 genes. Some individuals can have one of many causative alterations or two confound heterozygous recessive states. Hence, we need therapy pointed towards the molecular mechanism of the HSP genotypes associated with cellular structure, dysfunction, and altered pathways as it can be useful for multiple genotypes. The cellular functions related to HSP pathophysiology are linked to defects in the metabolism of lipid molecules, organelles morphology, endo-lysosomal functioning, and axons transportation [10]. Though the unravelling of the pathophysiological aspect of HSP is progressing continuously, the specific clinical management and cure are still lagging due to the overlapping similarity between the pathways and clinical manifestations with other neurological deteriorating diseases. So far, HSP management majorly includes physiotherapy, antispastic drugs, and botulinum toxin [10,11]. Hence, the current necessity is to find a cure or effective management therapy for HSP. Our present review sums up the available therapeutics for HSP treatment to date and other possible medicaments.

## 2. HSP Epidemiology at a Genetic Level

According to the global geographical location, the prevalence of HSP varies from 1 to 5 among 100,000 individuals, with the majority showing mutations in the *SPAST* gene in the autosomal dominant pure pattern in northern America and northern Europe [12]. The prevalence of familial inheritance is often higher than the sporadic form (2:1) in 70% of autosomal dominance linked to pure HSPs [13].

**Figure 1:**
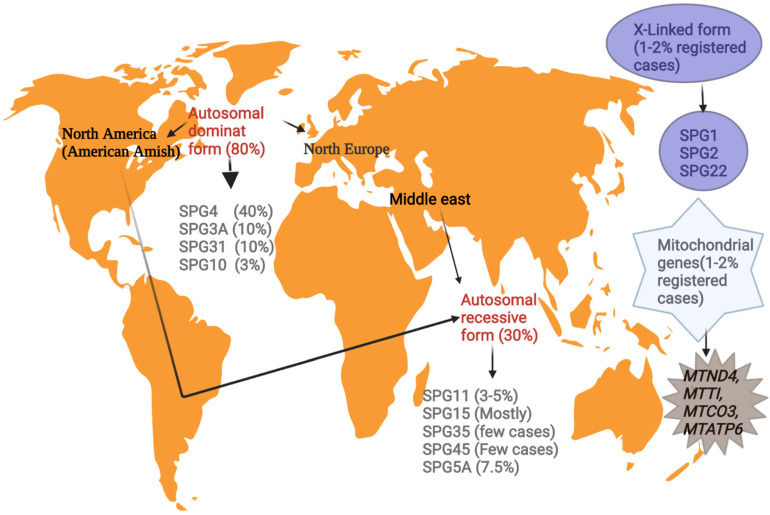
Illustrates the genetic epidemiology of the major HSP phenotypes around the world.

Nations with higher consanguinity are associated with the autosomal recessive HSP form in ethnicities such as Middle Eastern and American Amish groups (Figure 1). This pattern mostly appears in the early phase of life in HSP patients with multifaceted phenotypes. Mutations in the SPG11 gene are the highly prevalent autosomal recessive, followed by SPG15, SPG7, and SPG5 [7].

To date, three HSPs are observed to be X-chromosome-linked and four mitochondrial genes cause HSP with a prevalence rate of 1–2% in the registered HSP cases [12].

Alterations in “non-SPG” genes can also induce HSP characteristics in a single family. The advent of next-generation sequencing (NGS) led the complications in the genetic basis of HSPs and showed mixed inheritance patterns (both dominant and recessive) in six HSPs. The presence of one dominant inheritance pattern and other probable allele dose-dependent differences induces genetic heterogeneity and complexity. The *KIF1C* gene encoding clarifies allele dose-dependent variability for Kinesin. The *KIF1C* alteration shows a mild dominant phenotype in heterozygous cases, whereas a similar alteration in recessive phenotypes is observed in the homozygous state to induce SPG58 [10]. The pther mixed inherited variants with a limited correlation between phenotype and genotype are SPG7 and KIF1A [14].

## 3. Clinical Manifestations

The initial sign of HSP includes mild stiffness with or without the loss of muscle tone in the lower extremities. Additional cues to exclude from another spasticity-like disease include the age of onset, presence of familial history, and the progression of clinical features, as well as magnetic resonance imaging (MRI) of the brain to some extent [13].

The presence of diverse genetic markers implies the existence of a wide range of clinical manifestations, from distinct pyramidal signs in the legs, primarily involving the upper motor neurons, to a group of other motor neurons to trigger other neurological manifestations, such as peripheral neuropathy, cognitive disability, and cerebellar ataxias [15]. The pure form of HSP is observed in families with a wide range of disease severity such as walking disabilities, disturbed lower limb sensation, and urinary complications [13]. The degree of spasticity is disclosed by neurological tests in hamstrings, adductors, quadriceps, and gastrocnemius-soleus muscles leading to gait disturbances along with ankle dorsiflexion and hip flexion. Predominantly, progressive loss and weakness in the lower extremities are the common findings linked with the disability, and a hypertonic bladder results in urinary misfunctioning without affecting speech, cognition, and cranial nerves. The pure form of HSP can trigger at any age with slow progression over the years and elicit crossed reflexes of the adductor, extensor plantar, and ankle clonus generally [16,17]. Though individuals with the same mutations can show phenotypic variations or subclinical features, clinical manifestations can aid in differentiating the aetiology of the HSP pathology. The presence of both genetic and phenotypic heterogeneity makes HSP treatment challenging.

## 4. Cells Involved in HSPs Pathology

**Figure 2:**
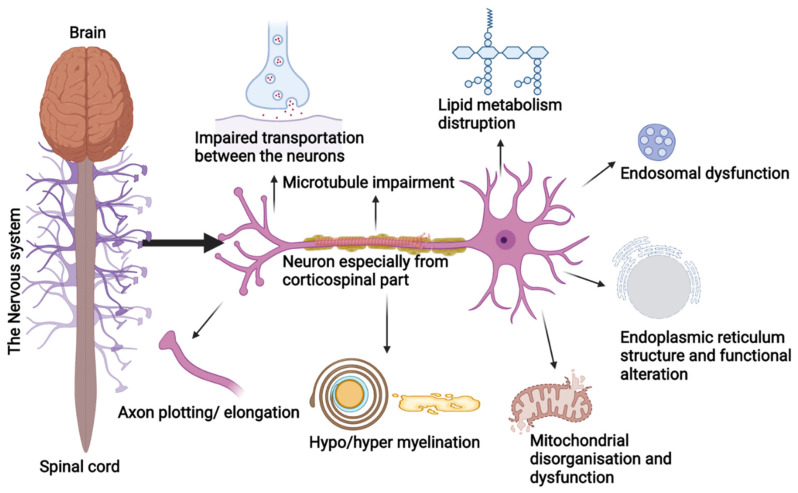
Schematic representation of the cellular process involved in the pathology of HSP.

Studies have shown that SPGs cluster around a few cellular functions and these proteins can appear in different pathways, which can lead to the evolution of pathogenic grouping in the coming time. Usually, HSPs are due to the degeneration of the longest neurons of the corticospinal tract, posterior columns, and spinal cord regions. These neurons are prone to organelles impairment and are involved in the transportation of macromolecules by consuming higher metabolites (Figure 2). Disruptions during cellular processes, organelle shaping, distribution, and trafficking are the prime cause of HSP pathologies [18]. The HSP genes are overlapped into the following functional groups:

### 4.1. Endoplasmic Reticulum (ER) Morphology and Network

ER is a distinct continuous membranous organelle with heterogenic morphology and presence throughout the cells. ER is involved in the synthesis, alteration, quality assessment, and trafficking of secretory proteins, integral membrane, and Ca^2+^ release, sequestration, and signaling. Especially in neurons, ER is involved in the membrane expansion occurring during axon and dendrite genesis by signaling Ca^2+^ release [19]. Mutations in the proteins linked to ER tubular network formation induce autosomal dominant forms such as SPG3A, SPG4, SPG31, and the less common SPG12 [20].

Additionally, ER tubules motility is organized with the help of microtubules (MT) sliding over ER for motor activity. Any ER and MT relationship impairment can trigger HSP pathology [20,21].

### 4.2. Axon Plotting

In the developing central nervous system (CNS), *L1CAM* is one of the major genes for HSP pathology, which interacts with neuropilin-1(Nrp1) followed by Plexin-A to form Semaphorin 3A (Sema 3A), required for steering away from the corticospinal neurons from the medullary or midline spinal cord. Mutations in the *L1CAM* gene hinder Sema3A functioning, while axons cross and lead to the early beginning of SPG1 [22].

### 4.3. Lipid Metabolism and Synthesis

The lipids and sterols are metabolized, distributed, and synthesized by the ER in both vesicular and non-vesicular modes to promote axon health and can be a perfect fit for the HSP pathogenic models. Proteins such as spartin and seipin are involved in the lipid droplets’ biogenesis, and alterations in the gene lead to Troyer syndrome or SPG20 and SPG17 [20,23].

Likewise, other genes such as *SLC33A1*/SPG42 acetyl-CoA are necessary for carbohydrates. Fat metabolism and its link in axonal growth, neuropathy target esterase *PNPLA6*/SPG39, essential for the generation of axons in the long spinal cord, and the *CYP7B1*/SPG5 gene play a fundamental role in the metabolism of cholesterol [20,24]. Additionally, genes involved in the synthesis and degradation of varied lipid classes are also involved in HSP phenotypes such as P450-7B1(CYP7B1), EPT1/Selenol, SERAC1, PLA2G6, GALC, and SLC33A1, and more genes are responsible for lipid metabolism [25].

### 4.4. Myelination

The abnormal myelination of the CNS neurons triggers HSP manifestations; alterations in the membrane’s *PLP1* gene of the integral proteolipid protein are commonly observed to induce SPG2. *PLP1* proteins comprise the major myelin component in the CNS [26]. Alteration in the connexin 47 (CX47) encoding gene *GJC2* leads to complicated SPG44 characteristics. CX47 connects oligodendrocytes and astrocytes and is required for myelination maintenance in the CNS [27]. Another dysmyelination-associated phenotype is SPG35, caused by mutations in the fatty acid 2-hydroxylase gene (*FA2H*) necessary for maintaining the galactolipids in the myelin sheath [28] as observed in animal models.

### 4.5. Impaired Motor Neurons Transport

The SPG10 phenotype is observed due to alterations in the *KIF5A* gene necessary for transporting motor information as these proteins move in an anterograde direction in the axons [29]. The movement of the cargoes in the dendrites and traffic pathways is regulated by the *KIF5* genes [30].

### 4.6. Endosomal Kinetics

The protein spartin, SPG20, interacts with the endosomal sorting complex required for the transportation (ESCRT) complex, and another protein, spastin, SPG4, harbours microtubule-interacting and trafficking proteins necessary for cytokinesis and vesicles sorting, along with epidermal growth factor signalling and degradation [31]. Another protein, strumpellin, SPG8, is a subunit of the Wiscott–Aldrich syndrome protein and suppressor of cAMP receptor homolog complex (WASH), necessary for generating the actin network in the tubular transportation of primitive endosomes [32]. WASH also communicates with the AAA ATPase of valosin, needed to maintain the frontal and temporal lobes, and its alteration can lead to neurological disorders such as HSPs [20].

Spastizin/SPG15, Spatacsin/SPG11, and KIAA0415/SPG48 are emerging proteins related to HSP phenotypes due to their participation in endocytic trafficking [33,34]. Endosomes are a key pathogenic model for HSP due to the growth of HSP gene lists related to endosomes functioning among the protein complexes. WASH activates actin networking on the endosome surface and aids endosome tabulation and sorting. Similarly, proteins involved in the HSP phenotype as a result of autophagy and lysosome dysfunction, such as spatacsin, AP5 complex, and spastizin, are found in endosomes.

### 4.7. Mitochondrial Dynamics

Clinical manifestations, such as neuropathies of the peripheral neural system and gait disturbances with visual and cognitive impairments, are linked with mitochondrial disorganization and dysfunction [33]. Paraplegin/SPG7 and heat shock protein HSP60/SPG13 are the mitochondrial proteins necessary for assembling ribosomes and the quality check of the proteins [34]. Impairment in the heat shock protein HSP60 chaperonin activity is linked to the late onset of HSP in its pure form due to dysfunctional mitochondrial activity [35]. Chaperonin resides in the mitochondria and maintains the protein homeostasis in the organelle. Mutations in heat shock protein lead to impaired protein folding, triggering SPG13 [36]. 

### 4.8. Microtubule Dynamics

Microtubules encourage the growth of axons in central nervous system neurons and help maintain the neurons’ morphology, transport, and motility, along with maintaining the complexity of neutrine arrangement and degradation. Spastin protein encoded by the *SPAST* gene is necessary for maintaining microtubule dynamics, and its mutation is linked with SPG4 [37]. Spastin serves microtubules in more stable regions by targeting tubulin post-translation [38,39]. 

## 5. Diagnosis

**Figure 3:**
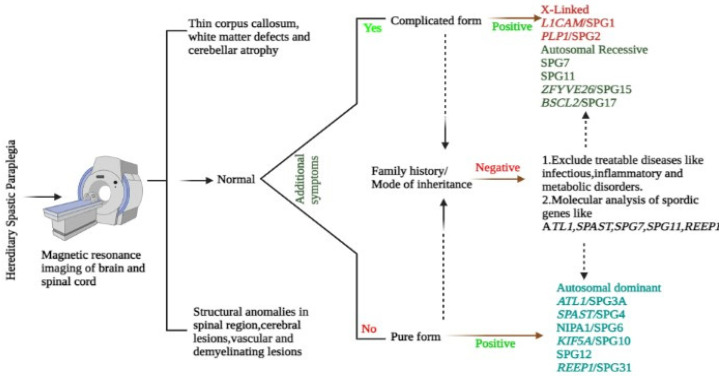
Elucidates the diagnostic protocol followed to identify HSP patients and other neurological disorders.

The HSP pathology is identified using the designated SPG gene mutation analysis molecularly, with the exclusion of acquired spastic paraparesis caused by structural and inflammatory infections plus Vitamin B12 or copper deficiencies. Cases of arteriovenous fistulas or amyotrophic lateral sclerosis should be excluded too [40] (Figure 3).

### 5.1. Genetic Testing 

Genetic panels are available cost-effectively worldwide to diagnose the HSP phenotypes through next-generation sequencing (NGS) of the exons linked to HSP. NGS has the limitations of not identifying the copy number variants such as exon deletions and duplications or the alterations in the promoter region, deep intronic region, and disorders such as triplet repeats [9]. In the case of normal sequence results, multiplex ligation-dependent probe amplification is performed, mostly in exon deletion cases for *SPAST* genes [41]. The clinicians are in charge of deciding the genes for multiple ligation assays to identify a variant of uncertain significance. In addition, clinicians should have well-versed knowledge about other monogenic disorders with clinical manifestations such as progressive lower extremities spasticity without imagining abnormality to exclude it from HSP categorization [40].

### 5.2. Magnetic Resonance Imaging (MRI)/Neuroimaging

Most of the SPG manifestations are linked with the thinning of the spinal cord, especially the rare X-linked SPG2, which is signified with a diffused hypomyelination pattern [42]. Only a few known pathognomonic alterations in the brain help to identify SPG types such as cerebellar atrophy in SPG7 from 39 to 95% [43,44]. Likewise, corpus callosum thinning and the intensification of periventricular white matter are linked to SPG11 and SPG15 [45]. 

### 5.3. Neurophysiology

Studies on SPG 4 by Martinuzzi and 11 Italian cohorts found absent or delayed central motor neuron conduction in the lower limbs [46]. Likewise, studies related to electromyography and conduction of neurons showed changes in neurogenic alterations in SPG7, SPG11, SPG15, SPG3A, SPG4, and SPG5 cases [47]. These changes can predict the predisposition characteristics for HSP subtypes.

## 6. HSP Treatment and Management

**Figure 4:**
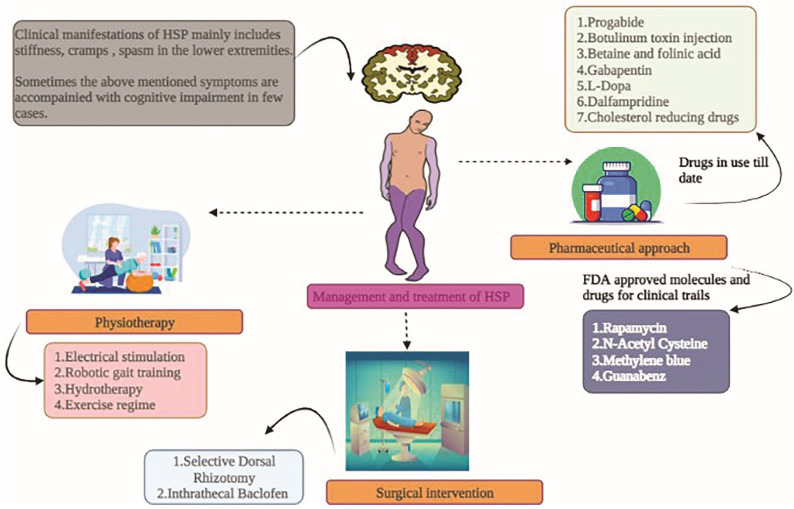
HSP patients have shown the clinical manifestations, and the treatment and management options available to date. The combination of physiotherapy and the pharmaceutical approach has been shown to provide good output. However, studies must be conducted on large cohorts to offer evident results to the management and therapeutic approach.

The fundamental approach for treating and controlling HSP addresses symptoms such as stiffness, deformities, spasms, and cramps (Figure 4). The exercise routine suggested by neuro-physiotherapists, primarily targeting stretches, followed by balance, is of prime importance. Below are the therapeutic approaches to tackling and subsiding the HSP pathophysiology (Table 1).

### 6.1. Physiotherapy

Very limited evidence, including uncontrolled studies and case reports, suggests the efficacy of physical therapies in HSP patients.

- **Electrical stimulation**

Only one case report has been published so far suggesting the efficacy of electrical stimulation in a 26-year-old man to improve motor neuron function. Quadriceps and anterior musculature bilateral stimulation thrice a week for three months improves the gait analysis performance result, and this case report is rated very low [54].

- **Gait training using robots**

Various robotic locomotive systems are currently utilized to improve the gait in patients with neurological disorders. Only one uncontrolled study has shown the beneficial effect of robotics help in intensive training among pure HSP-affected adults. The berg balance and Meter walk test scores were significantly improved with the robotic aided training [58].

One case report showed a slight improvement in walking speed and balance in a 28-year-old pure HSP man after giving 25 training sessions for six weeks with physiotherapy and robotic training [55].

- **Physical training**

To date, there is no significant evidence to support specific physical training exercises and timing for HSP cases. However, one study has reported the benefits of strengthening, stretching, and basic functional exercise in HSP adult siblings for 8 weeks daily/90 min. Due to a lack of data on the improvement in the precision parameters, conclusive statements cannot be provided at present [59].

- **Hydro-training**

A small uncontrolled trial has assessed the efficacy of hydro-training in enhancing the locomotor function in late-onset HSP patients after 10 weeks of 45-min sessions. Significant improvement was observed in the kinematics and spatiotemporal measures to improve gait. However, further studies are required to signify the efficacy of hydro-training [60].

### 6.2. Surgical Options

Since very few random uncontrolled trials exist, the efficacy of interventions and surgical options for HSP therapy is not significant and conclusive.

- **Selective Dorsal Rhizotomy (SDR)**

Selectively removing the troublesome nerves from the spinal cord benefits cerebral palsy patients. A similar procedure was carried out on four HSP adults and four children with HSP, and the results were evaluated after 6 and 12 months, followed by a yearly evaluation. Significant improvement in the lower extremities spasm score was observed without reappearing local complications. SDR can be implemented in patients with uncomplicated HSPs with spinal-cord-linked spasticity rather than in complicated cases [11].

- **Intrathecal delivery of Baclofen (ITB)**

The GABAB receptor activator baclofen is given intrathecally or orally for relaxing the muscles as an antispastic agent. The intrathecal mode is more effective and has less toxins than the oral mode. Only one study is available in a literature survey showing the efficacy of ITB in HSP cases at a mean dose of 90μg every day. ITB has been shown to reduce lower limb spasticity and improve the walking scale score [61]. Another study by Dan and Cheron [62] showed that administering ITB improved the electromyographic and Modified Ashworth Scale (MAS) score in HSP adults. Likewise, another study showed that ITB also decreased spasticity and retained muscle strength [56].

### 6.3. Pharmacological Therapeutics

Below, some drugs shown to have promising results in HSP treatments are mentioned. Other symptoms such as bladder dysfunction in HSPs are treated with oxybutynin or tolterodine [10].

- **Progabide**

The administration of a GABA agonist progabide with a median dose of 24.3 mg/kg significantly reduces hypertonia spasticity, flexor spasms, and reflex responses compared with placebo [11].

- **Injecting botulinum toxin**

In uncontrolled clinical trials with HSP cohorts containing SPG8, SPG4, SPG3A, and other autosomal dominant variants, injecting type A botulinum toxin (BoNTA) intramuscularly for spasticity was found to be effective. Injecting 500-750 MU BoNTA into the triceps surae in both arms for 18 weeks with stretching exercises resulted in increased comfortable gait velocity. Another study found a considerable improvement in baropodometric tests and MAS muscle tone metrics [11,52].

- **Betaine and folinic acid**

According to literature studies, complicated HSP patients with methylenetetrahydrofolate reductase inadequacy treated with vitamins B12 (1000 mg per month), folinic acid (45 mg per day), folic acid (15 mg per day), and betaine anhydrous (10 g per day) resulted in the reduction in homocysteine concentration and improved the conditions. However, more studies are required to obtain conclusive data [11].

- **Gabapentin**

Gabapentin inhibits the calcium wave by activating the activation of the α2d-1 subunit required to lower neurotransmitter conduction and weaken the postsynaptic excitation. Tests on a HSP group for two months with 2400 mg per day followed by a free 1-month drug-free interval showed no statistically significant results compared with a placebo group. The study was observed in a small group of 10 members, so the conclusive output cannot be comprehended [63].

- **L-Dopa**

This dopamine precursor is shown in HSP patients with improved motor neuron symptoms and spasticity in SPG11 cases, those with thin callosum, and SPG8 subjects. About 300 mg daily is administered to observe the patients’ response to biallelic mutations for SPG11. An anecdotal study assessing the drug’s efficacy must be studied in large cohorts [11].

- **Dalfampridine (4-Aminopyridine)**

4-Aminopyridine is used as a potassium channel blocker in neurological patients to improve walking ability. Oral administration of dalfampridine (10 mg) twice continuous for 15 days showed significant enhancement in the Spastic Paraplegia Rating Scale (SPRS) and the 12-item Multiple Sclerosis Walking Scale (MSWS-12) [49]. Likewise, the MAS assessment was also improved in another study comprising SPG4 and SPG15 patients with the same oral dose of dalfampridine. These studies were conducted in small groups, and limited further controlled studies are required to ascertain the efficacy of dalfampridine [50].

- **Cholesterol decreasing therapeutics**

HSPs, such as SPG5, are due to mutation in the oxysterol 7α-hydroxylase gene *CYP7B1* causing the stagnation of oxysterol neurotoxins. Treating randomized controlled groups with 20 to 60 mg per day with cholesterol-decreasing drugs such as simvastatin for a year and ezetimibe and simvastatin in the following year showed a validated decrease in the SPG5 (27-hydroxyoxysterol) marker in the patient’s serum [11]. This study is a preliminary report on the efficacy of this drug in SPG5 cases in the long term.

### 6.4. Food and Drug Administration (FDA)-Approved Therapies for Clinical Trials

A few drugs, such as Rapamycin (RM), N-Acetyl Cysteine (NAC), Guanabenz (GA), and Methylene blue (MB), have shown good scope in the treatment of neuronal dysfunction in animal models (Table 2). 

## 7. Scope and Future of Therapeutics

So far, no proper medicinal treatment is available for HSP phenotypes; hence, finding the primary drug target for all the HSP subtypes is the need of the hour. As all the cellular pathological themes are overlapping and intertwined, lipid droplet formation is required for trafficking and signaling the membranes, which depends on the adenosine triphosphate (ATP) production from the mitochondria. These suggest the varying pathways’ functional results, making it theoretically possible to find a promising drug for the wide range of HSP subtypes. However, HSP practically probands the alteration of many pathways, making common drug discovery difficult.

Additionally, the lack of available biomarkers makes drug curation more arduous. However, these little-known biomarkers help monitor the progression and treatment response in the preclinical and clinical trials among animals. Few laboratory findings and non-clinical diagnostics, such as hormonal alteration, biochemical parameter alteration, or piling up of metabolites, can aid drug discovery. Certain parameters such as 25- and 27-hydroxycholesterol enhancement are observed in the cerebral spinal fluid (CSF) and blood of patients with SPG5 [64]. Additional parameters include higher levels of testosterone in SPG46 male blood [65], increased concentration of plasmatic amino acids in SPG9 cases [66], irregular ether lipid profile among SPG82 [67], volumetric alteration in brain images of SPG11 [68], and SPG56 patients showing brain calcification along with the presence of neopterin (Coenzyme Q10) [69]. Lipid level alteration is observed in SPG11, as lipid metabolism is necessary for the cellular function of neurons [70]. Drugs targeting the regulation of microtubules are beneficial for models of SPG4 and SPG3 [71]. Advancing pathogenic research and increasing approaches toward personalized gene therapy are lagging in cases of HSPs due to limited data and trials being conducted on small groups of HSP patients. To evaluate the efficacy of the already used and FDA-approved drugs, studies must be performed on a larger sample size to obtain precise significant biological outcomes.

## 8. Conclusions

### Hinge between Molecular Mechanisms and the Clinical Aspects

HSP diagnosis is still crucial to identify the genetic aetiology of the pathology to provide the person with a conclusive description of the long, wandering diagnostics, further helping to counsel the patient and, above all, helping to identify the mechanism underlying the HSP pathogenicity. With proper genetic knowledge, better treatment options would be available and help delineate the HSP’s heterogeneity. Additionally, identifying the genes and pathways will aid in identifying appropriate biomarkers and the predictors of early onset of the pathology, along with the guide for a treatment option to provide a good quality of life.

Though the efficacy and sensitivity of genetic testing have been improved over the years with the utilization of NGS with multiple gene testing panels in the field of HSPs, clinical awareness is still required due to a high number of full mutations revealed by NGS, called significant variables of uncertainty (>200). Two decades back, the cloning of the first SPG has given insight into the HSP pathophysiology and feeds the hope of finding effective therapeutics through the cooperation of geneticists, clinical neurologists, and molecular biologists. Currently, the hype related to the underlying molecular mechanism of HSP is at its peak, so it will be challenging to accommodate the new findings in clinical practices in the coming days. The hope of identifying novel genetical strategies at molecular levels to predict the pathology at the earliest stage in carriers and patients can make the diagnosis and management comparably easier. Complex multifaced pathogenic pathways imply the absence of a “one bullet” kind of therapy. Rather, a combination of therapeutic modes is required for relaxing and improving the tone in hypercontractive muscles.

In a nutshell, HSP patients or carriers of the mutant genes will be given individualized therapy based on the neurotrophic factors such as neurite lengths, branching, swelling obtained from their brain, a regime of physiotherapy, and the epigenetic surrounding of the individuals. Based on this view, the classification of HSP should rely on the pathophysiological mode rather than clinical manifestation alone. Specific subtypes with the matching genotype of HSP patients can open gates to dive deeper into the underlying molecular cause of HSPs to further achieve personalized medicine therapies. Gene therapy might be beneficial as it can analyze the biochemical pathway and provide an opportunity for replacing or editing the target gene in trail-ready cohorts. Resource pooling and developing therapeutics in HSP will be beneficial in the coming days.

## Figures and Tables

**Table 1:** An overview of the therapeutic and surgical approachs used in HSP management to date and the quality of the protocol used (Good—efficacious; Moderate—obtained substantial results from large cohorts; Poor—requires more clinical trials in large cohorts to ascertain the impact).

Therapeutic Approach	Type of Study	Quality Grade
**Pharmaceutical drugs**		
Gabapentin	A prospective double-blinded study between placebo and control [48]	Good
Progabide	A prospective double-blinded study between placebo and control [49]	Moderate
Dalfampridine	Uncontrolled prospective open trial study [50]	Poor
Botulinum toxin	A retrospective study [51]	Poor
L-Dopa	Case report [52]	Poor
Betaine and folic acids	Case report [53]	Poor
**Physiotherapy**		
Electrical stimulation	Case report [54]	Poor
Robotic gait training	Uncontrolled prospective trials and case reports [55]	Poor
Hydrotherapy	Uncontrolled future trails [1]	Poor
Exercise regime	Case report [2]	Poor
**Surgical interventions**		
ITB	Open uncontrolled studies, case report [56]	Poor
SDR	Retrospective study [57]	Poor

**Table 2:** Illustrates the chemical structure of the molecules and drugs, which can plausibly manage and cure HSP. The FDA approves these drugs to conduct clinical trials as they have shown positive results in animal models.

S. No	FDA Approved Drugs	Chemical Structure
1	Rapamycin	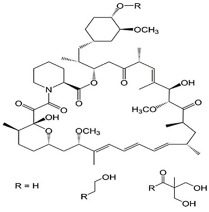
2	Methylene blue	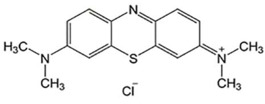
3	N-acetyl-cysteine	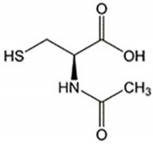
4	Guanabenz	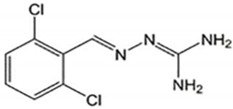

The FDA has approved these drugs to be used in control studies to know about their conclusive effects in HSP pharmacology [10].

## Data Availability

Not applicable.
